# A Blended Learning System to Improve Motivation, Mood State, and Satisfaction in Undergraduate Students: Randomized Controlled Trial

**DOI:** 10.2196/17101

**Published:** 2020-05-22

**Authors:** Mario Lozano-Lozano, Carolina Fernández-Lao, Irene Cantarero-Villanueva, Ignacio Noguerol, Francisco Álvarez-Salvago, Mayra Cruz-Fernández, Manuel Arroyo-Morales, Noelia Galiano-Castillo

**Affiliations:** 1 Department of Physical Therapy University of Granada Granada Spain; 2 Sport and Health University Research Institute University of Granada Granada Spain; 3 Biohealth Research Institute in Granada Granada Spain; 4 Departamento de Lenguajes y Sistemas Informáticos e Ingeniería de Software Universidad Politécnica de Madrid Madrid Spain; 5 Scientific Unit of Excellence on Exercise and Health University of Granada Granada Spain

**Keywords:** learning, teaching, students, health occupations, mobile apps, education

## Abstract

**Background:**

Smartphone-based learning, or mobile learning (m-learning), has become a popular learning-and-teaching strategy in educational environments. Blended learning combines strategies such as m-learning with conventional learning to offer continuous training, anytime and anywhere, via innovative learning activities.

**Objective:**

The main aim of this work was to examine the short-term (ie, 2-week) effects of a blended learning method using traditional materials plus a mobile app—the iPOT mobile learning app—on knowledge, motivation, mood state, and satisfaction among undergraduate students enrolled in a health science first-degree program.

**Methods:**

The study was designed as a two-armed, prospective, single-blind, randomized controlled trial. Subjects who met the inclusion criteria were randomly assigned to either the intervention group (ie, blended learning involving traditional lectures plus m-learning via the use of the iPOT app) or the control group (ie, traditional on-site learning). For both groups, the educational program involved 13 lessons on basic health science. The iPOT app is a hybrid, multiplatform (ie, iOS and Android) smartphone app with an interactive teacher-student interface. Outcomes were measured via multiple-choice questions (ie, knowledge), the Instructional Materials Motivation Survey (ie, motivation), the Profile of Mood States scale (ie, mood state), and Likert-type questionnaires (ie, satisfaction and linguistic competence).

**Results:**

A total of 99 students were enrolled, with 49 (49%) in the intervention group and 50 (51%) in the control group. No difference was seen between the two groups in terms of theoretical knowledge gain (*P*=.92). However, the intervention group subjects returned significantly higher scores than the control group subjects for all postintervention assessed items via the motivation questionnaire (all *P*<.001). Analysis of covariance (ANCOVA) revealed a significant difference in the confusion and bewilderment component in favor of the intervention group (*P*=.01), but only a trend toward significance in anger and hostility as well as total score. The intervention group subjects were more satisfied than the members of the control group with respect to five out of the six items evaluated: general satisfaction (*P*<.001), clarity of the instructions (*P*<.01), clarity with the use of the learning method (*P*<.001), enough time to complete the proposed exercises (*P*<.01), and improvement in the capacity to learn content (*P*<.001). Finally, the intervention group subjects who were frequent users of the app showed stronger motivation, as well as increased perception of greater gains in their English-language competence, than did infrequent users.

**Conclusions:**

The blended learning method led to significant improvements in motivation, mood state, and satisfaction compared to traditional teaching, and elicited statements of subjective improvement in terms of competence in English.

**Trial Registration:**

ClinicalTrials.gov NCT03335397; https://clinicaltrials.gov/ct2/show/NCT03335397

## Introduction

Smartphone-based learning or mobile learning (m-learning), a term that highlights the type of device used, has become a popular learning-and-teaching strategy. It has been defined as the ability to access educational resources, tools, and materials anytime and from anywhere, using a mobile device, such as a smartphone [1]. With respect to health professionals and their education, Dunleavy et al describe m-learning as “any intervention using handheld, mobile devices connected through wireless connections to deliver educational content to pre and postregistration health professionals in order to extend the reach of learning and teaching beyond physical space and distance” [2]. M-learning thus provides a self-directed learning environment that affords continuous access to knowledge, information, and practice tools.

M-learning is being increasingly used by undergraduate and postgraduate students undertaking specialty or continuous training in the health sciences [2,3]. A recent review and meta-analysis showed that m-learning is equally or more effective than traditional learning in this field, although the authors also comment on the need for further research into its value [2]. Given the rapid evolution of mobile technologies in general, and of m-learning in particular, up-to-date evidence is required for the effectiveness of these strategies to be determined.

Motivation is an important factor in learning and performance [4]. Motivation levels can vary depending on learning-and-teaching style. A positive relationship has been reported between motivation and academic performance and learning, while a negative association is associated with dropping out from education [5]. Thus, motivation can be increased if the learning-and-teaching style is appropriate [6,7]. M-learning incorporates different activity options, which students can choose depending on their personal learning preferences or circumstances. This can encourage motivation, engagement, and learning success, positively influencing mood status [6,7].

New technologies may, however, also have a negative effect on learning. A recent study [8] highlights how students believed that new technologies in the classroom might affect their concentration and ability to learn. However, other investigations have found that students using electronic devices during a lecture returned results in learning that were similar to those who did not use these devices [9]. In other work, using electronic devices as learning tools while listening to a teacher was reported to not be distracting [10]. Clearly, the impact of the use of m-learning needs to be further investigated.

Blended learning that combines m-learning with conventional learning to seek the benefits of both [11,12] can also improve the educational experience [13], allowing conventionally taught material to be revisited whenever and wherever the student wishes [12,14]. Previous investigations into blended learning have returned positive results [15,16]. The main aim of this work was to examine the short-term effects of a blended learning method using traditional materials plus a mobile app on specific outcomes among students enrolled in a health science first-degree program. It was hypothesized that, compared to traditional learning alone, a 2-week blended learning program would improve knowledge uptake, student motivation, mood, and satisfaction and would improve competence in English.

## Methods

### Study Design and Sample Size

This study was a two-arm, prospective, single-blind, randomized controlled trial (ClinicalTrials.gov identifier: NCT03335397). The study subjects were 99 students in their first year of a health sciences degree at the Faculty of Health Sciences, University of Granada, Spain.

The sample size calculation was based on a previous study [17] and a demand of 90% power to detect a difference of 1.70 points in knowledge gain (see Main Outcomes section), while assuming a type 1 error (alpha) of 5% and a type 2 error (beta) of 10%, determined using G*Power software, version 3.1.9.3 (Heinrich-Heine-Universität Düsseldorf), for Mac OS X [18]. The required sample size was estimated at 40 subjects each for the intervention and control groups. Contemplating a dropout rate of 25%, as it is common for health science students to migrate to medicine in the first semester of their first academic year, 50 subjects were enrolled per group.

### Recruitment

Subjects were recruited via a talk given on the first day of the course unit entitled Basic Health Science, a unit lasting one semester. It was emphasized that no assessment made during the trial would have any effect on final grades and that participation was totally voluntary. To be included, the subjects had to (1) possess basic skills in handling mobile apps, (2) have a smartphone running either Android operating system (OS) or iOS software, (3) install the iPOT mobile learning app, (4) be enrolled in the above course unit, and (5) have a basic knowledge of English, accredited or not. In addition, all subjects had to provide informed consent to be included. Students repeating the unit were excluded.

### Randomization

**Figure 1:**
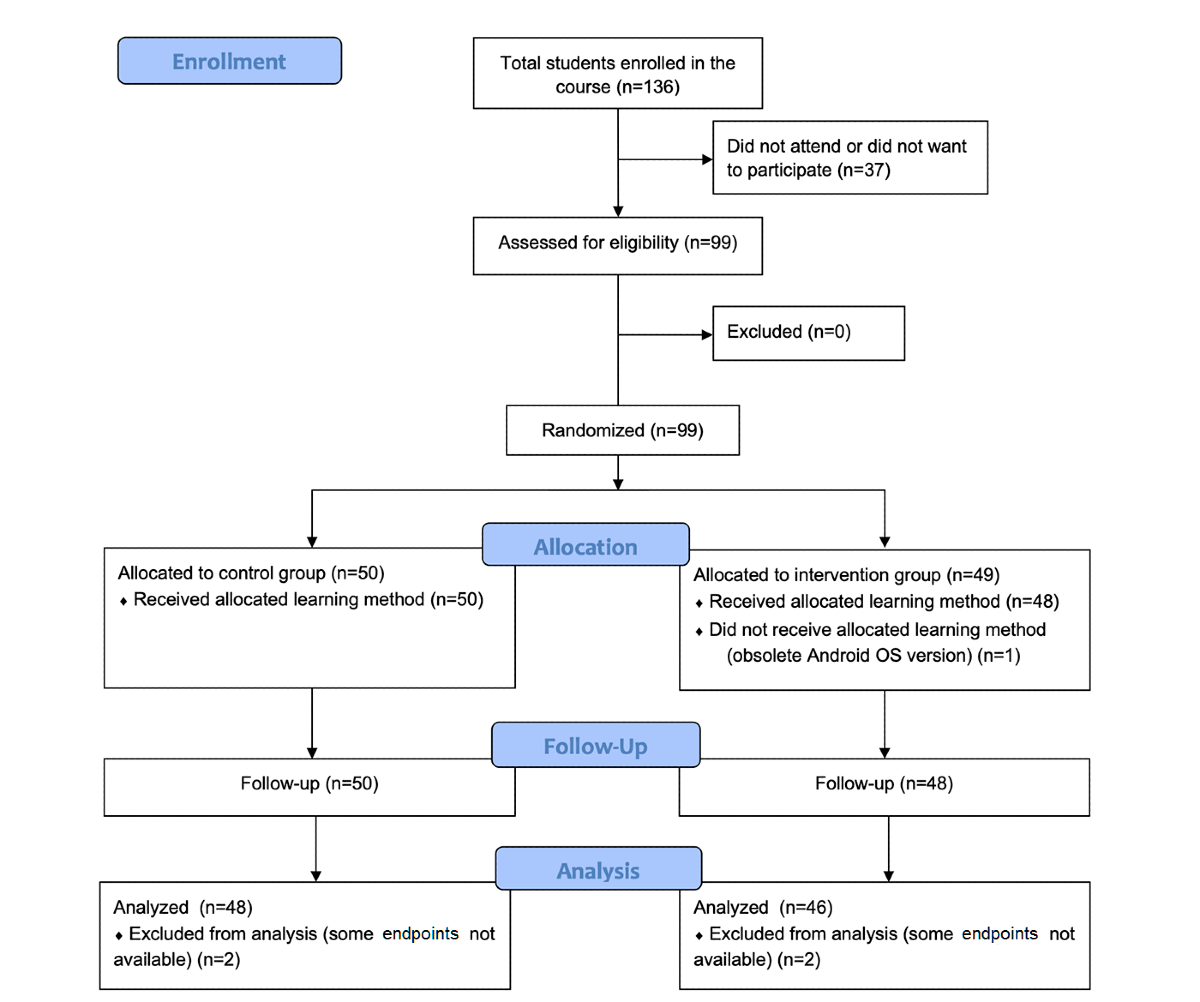
Flow diagram of the recruitment and randomization process. OS: operation system.

Subjects who met the inclusion criteria were randomly assigned to either the intervention group (ie, blended learning, involving traditional learning plus m-learning via the use of the iPOT app) or the control group (ie, traditional on-site learning). Figure 1 shows a flowchart of the recruitment and randomization process. Subjects were allocated to these groups via the computer generation of a random number list and the subsequent production of a subject allocation sequence using SPSS statistics software, version 22.0 (IBM Corp). The subjects then met with the evaluator who would conduct the forthcoming assessments; this evaluator was blinded to all group assignments.

### Experimental Procedure

This study was conducted over 2 weeks in the first semester of the 2017-2018 academic year. For both groups, the educational program involved 13 lessons on basic health science. The subjects of both groups were progressively introduced to conventional learning materials (ie, books, PowerPoint presentations, and journals available in the university library). The subjects in the intervention group, however, also received the iPOT app to reinforce the educational program; it provided no additional material. Both the intervention and control subjects were free to reinforce the taught information using any of the traditional sources available. All subjects in the intervention group received a QR (Quick Response) code to provide them access to the Apple App Store [19] or the Google Play store [20] as required. Subjects then installed the iPOT app. A lecturer involved in the project was present at this time to help solve any technical problems that might arise. All the intervention group subjects were provided a personal password linked to their institutional email account that allowed them to access the app, but were also assured each person's log-in information remained confidential and nonexchangeable with subjects in the control group; the system itself was enabled to detect irregular connections. However, given that students in the control group might borrow the devices of the intervention group members in order to try the app out, all subjects were requested to respect the protocol and told they would have unlimited access to the app at the end of the study. The iPOT app included a permanently available video tutorial (see Multimedia Appendix 1), accessible through the *Help* tab, to assist users in familiarizing themselves with the app and its operation. In addition, a WhatsApp group was established for the 2 weeks of the experimental period in order to resolve any incidents that might arise. Two smartphones were available to loan to students who wanted to participate but who had devices with compatibility problems.

M-learning lessons were enabled in the app over the last 2 weeks of the teaching period, allowing the intervention group subjects to review the information previously taught. Students were instructed to review each lesson via the six learning modalities available—quizzes (multiple-choice questions), alphabet soup (word search), hangman, hieroglyphics, puzzles (word sorting), and word-coupling activities—and were encouraged to use them; differences in use time were accounted for (see The iPOT Mobile Learning App: Internal Design and Technical Specifications section). It is important to highlight that during these 2 weeks, the control group subjects were encouraged to review the taught material via the above-mentioned books, PowerPoint presentations, and journals.

### The iPOT Mobile Learning App: Internal Design and Technical Specifications

**Figure 2:**
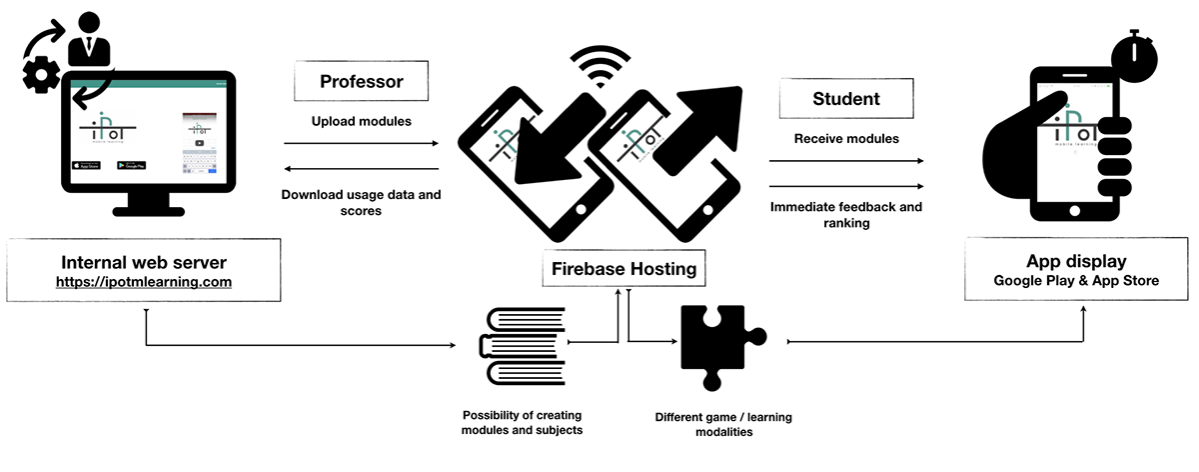
Top-level view of the iPOT app system.

The iPOT app was designed by a research group composed of lecturers in health science and an internship student of computer engineering, all at the University of Granada. The app is a hybrid, multiplatform (iOS and Android), smartphone app with an interactive teacher-student interface (see Figure 2). Its architecture involves Firebase, a Google cloud-based development platform that handles user authentication, file storage, and database management. The app interface gives users access to different game modes and allows them to complete different activities and find platform-related information. The app also has an administration console, developed using Angular and Angular Material development tools (Google), that gives administrators the possibility of managing users, all app modules, game-related data, and graphic resources by adding an abstraction layer between the administrator and Firebase. For the deployment of the administration console, it was decided to use Firebase Hosting, a static, rapid hosting service that includes a Secure Sockets Layer (SSL) by default, ensuring safe access.

The user interface shows the user profile, allowing access to personal data and providing the opportunity to change one's nickname; ranking (ie, position in terms of game scores; designed to help increase motivation via a sense of competition); and a start button to allow users to begin playing. As an incentive, each time a correct answer was given in any of the app's modalities, points were added to the user's score. Each user's scores, as well as the number of modules completed, were recorded via the system.

### Outcome Measures

Knowledge and mood state were assessed for both the intervention and control groups before and after the 2-week experimental period. Motivation, satisfaction, and English-language competence—the latter only measured in the intervention group—were assessed after this period.

#### Knowledge

Theoretical knowledge was evaluated at baseline (ie, the beginning of the 2-week study period) and at the end of the study period using 20 multiple-choice questions [21]. This tool has been used in other educational studies [22,23]. Scores were recorded on an 11-point scale, ranging from 0 to 10, with higher values representing better outcomes. This test was developed by two external lecturers who were not involved in the study.

#### Motivation

Motivation was assessed using the Instructional Materials Motivation Survey (IMMS) [24]. The terminology used by the tool was adapted to the learning methods employed in this study. The survey can be used to estimate the motivation and attitudes of students in situations of self-directed learning (ie, printed, virtual, or online courses). It contains 36 questions in four domains: attention, relevance, confidence, and satisfaction. Each question is scored on a 5-point Likert scale; therefore, total scores range from 36 to 180. Higher scores denote higher levels of learning motivation. The tool has been shown to be highly reliable (intraclass correlation coefficient [ICC]>.96) [25] and has been previously used in Spanish populations [26,27].

#### Mood State

Mood is a short-term state of feeling; it may fluctuate within minutes or over days. Unlike emotions, moods are more transient, often unrelated to external events, and are of varying intensity [28]. Subject mood state was assessed via the Profile of Mood States (POMS) questionnaire [29], validated for use in Spain [30]. This self-reported survey contains 65 questions, each scored on an increasing 5-point Likert scale, ranging from 0 to 4, that together measure six mood components: (1) tension and anxiety (nine items that refer to an increase in musculoskeletal tension, somatic tension, and observable psychomotor manifestations); (2) depression and melancholy (15 items that reflect a depressed state along with a feeling of sadness or guilt); (3) anger and hostility (12 items that represent feelings of anger and antipathy toward others); (4) vigor and activity (eight items that allude to a state of euphoria or high energy); (5) fatigue and inertia (seven items that refer to a mood of depression or a low level of exercise); and (6) confusion and bewilderment (seven items referring to disorientation or multiplicity of thought). The total average score of all the components (ie, the Total Mood Disturbance [TMD] score) was recorded. There are no cutoff points in any domain. Subjects were asked to fill in the questionnaire indicating how they felt lately. Lower values indicate a better general mood state [31]. The test is reliable; the ICC is close to .90 for all six components [29].

#### Satisfaction

Subjects' satisfaction with the learning methods to which they were exposed was evaluated via a questionnaire based on that reported by Brewer et al [32], with six items that assess satisfaction variables: general satisfaction, the clarity of instructions, whether the final assessment reflected the course syllabus, clarity with the use of the learning method, whether there was enough time to complete the proposed exercises, and improvement in the capacity to learn content. Each item was scored on an increasing 5-point Likert scale, ranging from 1 to 5. Higher values represent better outcomes. Subjects were also asked to report the number of hours per day, as well as the number of days per week, that they used the app and which mode of the app they preferred.

#### Linguistic Competence

The subjective improvement in English-language competence (ie, a general feeling of improvement and of improvements in written comprehension, written expression, vocabulary, and global perception) was measured at the end of the study via an ad hoc questionnaire, scoring answers on an increasing 5-point Likert scale, ranging from 1 to 5. Higher values represent better outcomes. To determine the subjects' prior English-language competence, they were asked about any official English certificates they possessed before starting the study.

### Statistical Analysis

The normal distribution of data was verified using the Kolmogorov-Smirnov test. Results are presented as mean (SD) values. The Student *t* test and Mann-Whitney U test or the chi-square test and Fisher exact test were used as required to examine differences in continuous and categorical variables, respectively, between the two groups (eg, sociodemographic characteristics, knowledge, and mood state) and in postintervention differences (eg, motivation, satisfaction, and English-language competence).

Differences between the groups in terms of the preintervention-to-postintervention change in knowledge and mood state were analyzed by repeated-measures analysis of covariance (ANCOVA), adjusting for the effects of those variables showing significant differences between groups at baseline (ie, the covariate *POMS-vigor and activity*). Missing values were few (<5% of the total number); in such cases, these can be considered *missing at random* and inconsequential [33]. For this reason, a list-wise deletion method was chosen; no multiple imputation was necessary. Significance was set at *P*<.05. All calculations were performed using SPSS statistics software, version 22.0 (IBM Corp), and Stata statistical software, release 14 (StataCorp).

### Ethical Approval

The study was performed in accordance with the ethical standards of the appropriate national and institutional research committees, and adhering to the Declaration of Helsinki [34]. Ethical approval was granted as required by Spanish Law 223/2004, of February 6, establishing subject confidentiality under the terms of Spanish Law 3/2018, of December 5 [35]. The study was also approved by the Quality, Innovation and Planning Unit of the University of Granada, Spain (Plan FIDO UGR 2016-2018). For ethical reasons, once the experimental phase of the project was complete, the app was made available to all subjects who took part.

## Results

### User Statistics

A total of 99 students were enrolled, with 49 (49%) in the intervention group and 50 (51%) in the control group. The mean age of the students was 19.76 years (SD 2.74) in the intervention group versus 20.00 years (SD 3.98) in the control group (*P*=.97). A total of 38 of the 49 subjects (78%) in the intervention group were women compared to 35 out of 50 subjects (70%) in the control group (*P*=.39). A total of 37 of the 49 subjects (76%) in the intervention group used mobile phones with the Android OS compared to 31 out of 50 subjects (62%) in the control group (*P*=.14). No significant differences were seen between the members of the two groups in terms of their baseline levels of English (*P*=.69) or baseline knowledge of the material to be taught (*P*=.43) (see Table 1). English levels were determined according to the Common European Framework of Reference for Languages: Learning, Teaching, Assessment [36]. The intervention group lost one subject (1/49, 2%) to follow-up—a dropout due to the subject's phone running an old OS that did not support the iPOT app—leaving a final initial sample size of 48. Another 2 subjects were lost from both groups due to incompletion of the final assessments (see Figure 1).

**Table 1:** Sociodemographic characteristics of the study subjects at baseline: the beginning of the 2-week study period.

Characteristic		Control group (n=50)	Intervention group (n=49)	
Age (years), mean (SD)		20.00 (3.98)	19.76 (2.74)	
**Gender, n (%)**				
	Male	15 (30)		11 (22)
	Female	35 (70)		38 (78)
**English level, n (%)^a^**				
	Not certified	25 (50)		22 (45)
	A2 (elementary)	4 (8)		2 (4)
	B1 (low intermediate)	14 (28)		15 (31)
	B2 (high intermediate)	5 (10)		9 (18)
	C1 (advanced)	2 (4)		1 (2)
**Mobile system, n (%)**				
	iOS	19 (38)		12 (24)
	Android operating system (OS)	31 (62)		37 (76)
Baseline knowledge of subjects (score^b^), mean (SD)		4.38 (1.21)	4.57 (1.22)	

^a^English levels were determined according to the Common European Framework of Reference for Languages: Learning, Teaching, Assessment [36]. A2: Basic User, Waystage; B1: Independent User, Threshold; B2: Independent User, Vantage; C1: Proficient User, Effective Operational Proficiency.

^b^Scores ranged from 0 (no knowledge) to 10 (highest level of knowledge).

### Main Outcomes

At the end of the experimental period, no significant difference was seen between the two groups in terms of theoretical knowledge gain (intervention group mean score 0.51 [SD 1.19] vs control group mean score 0.49 [SD 1.10]; *F*_1_=0.008, *P*=.92) (see Table 2). A significant difference was also not seen in the end-of-study total scores (mean score 5.16 [SD 1.30] vs mean score 4.90 [SD 1.24], respectively; t_92_=-1.019, *P*=.31). The covariate *POMS-vigor and activity* did not influence the results.

Table 3 shows that the intervention group subjects returned significantly higher scores than the control group subjects for all items assessed postintervention by the IMMS (IMMS_attention: t_92_=–12.223; IMMS_relevance: t_92_=–8.315; IMMS_confidence: t_92_=–7.731; and IMMS_satisfaction: t_92_=–10.631; *P*<.001 for all). Their IMMS total motivation scores were also significantly different (mean 136.02 [SD 19.25] vs mean 84.94 [SD 25.37], respectively; t_92_=–10.963, *P*<.001).

Table 4 reflects the mood state of the subjects in each group before and after the intervention. ANCOVA revealed a significant difference between the change in confusion and bewilderment for the control and intervention group subjects (mean score 3.67 [SD 8.66] vs mean score –0.50 [SD 6.62], respectively; *F*_1_=6.826, *P*=.01). A trend toward significance was also seen between the groups in terms of the change in anger and hostility (control group mean score 2.21 [SD 7.97] vs intervention group mean score –1.07 [SD 8.45]; *F*_1_=3.735, *P*=.05) and total score (control group mean score –1154.17 [SD 3652.10] vs intervention group mean score –17.39 [SD 2767.05]; *F*_1_=2.875, *P*=.09). No significant differences between groups were seen in terms of the change in tension and anxiety (*F*_1_=1.964, *P*=.16), depression and melancholy (*F*_1_=0.983, *P*=.32), vigor and activity (*F*_1_=0.181, *P*=.67), and fatigue and inertia (*F*_1_=0.233, *P*=.63). Adjustment for the covariate POMS-vigor and activity in ANCOVA revealed this variable to influence the change in confusion and bewilderment (*F*_1_=7.889, *P*=.006), anger and hostility (*F*_1_=4.493, *P*=.03), and total score (*F*_1_=4.603, *P*=.03). The covariate did not influence the rest of results.

**Table 2:** Effect of the different learning methods on knowledge gain.

Answers at study time points		Control group (n=48), mean (SD)	Intervention group (n=46), mean (SD)	*P* value^a^
**Correct answers**				.92
	Baseline^b^	8.81 (2.43)	9.30 (2.37)	
	Postintervention	9.79 (2.48)	10.33 (2.61)	
	Difference	0.98 (2.20)	1.02 (2.37)	
**Wrong answers**				.92
	Baseline	11.19 (2.43)	10.70 (2.38)	
	Postintervention	10.21 (2.48)	9.67 (2.61)	
	Difference	–0.98 (2.20)	–1.02 (2.37)	
**Total score**				.92
	Baseline	4.41 (1.21)	4.65 (1.19)	
	Postintervention	4.90 (1.24)	5.16 (1.30)	
	Difference	0.49 (1.10)	0.51 (1.19)	

^a^Repeated-measures analysis of covariance (ANCOVA) was used to examine the differences between groups.

^b^*Baseline* refers to the beginning of the 2-week study period.

**Table 3:** Learning motivation in the intervention and control groups.

Learning motivation variable^a,b^	Control group (n=48), mean (SD)	Intervention group (n=46), mean (SD)	*P* value^c^
Attention (12-60)	26.06 (9.64)	47.59 (7.20)	<.001
Relevance (9-45)	24.06 (6.58)	34.17 (5.08)	<.001
Confidence (9-45)	22.04 (6.96)	31.83 (5.13)	<.001
Satisfaction (6-30)	12.77 (4.55)	22.44 (4.25)	<.001
Total IMMS^a^ score (36-180)	84.94 (25.37)	136.02 (19.25)	<.001

^a^Learning motivation was measured using the Instructional Materials Motivation Survey (IMMS).

^b^Each category is presented with the possible range of its score in parentheses.

^c^The Student *t* test was used to examine the differences between groups. Significance was set at *P*<.05.

**Figure 3:**
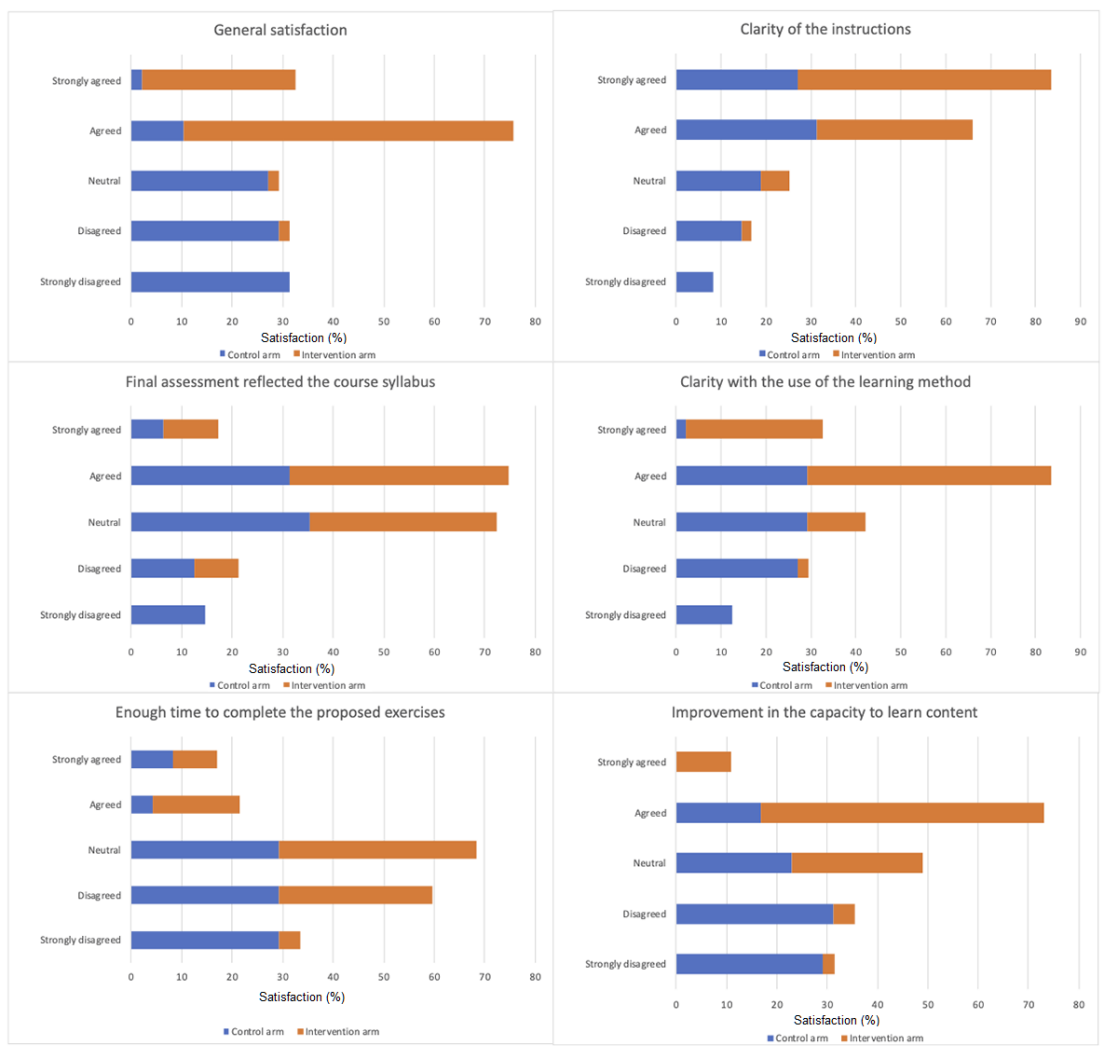
Satisfaction among the intervention and control arms.

Figure 3 shows the results of the global satisfaction survey with the learning method used. The intervention group subjects were more satisfied than the members of the control group in terms of all items evaluated: general satisfaction with the learning method (*Strongly agreed* and *Agreed* in the intervention group were 30% and 65%, respectively, vs 2% and 10% in the control group; *P*<.001), clarity of the instructions (*Strongly agreed* and *Agreed* in the intervention group were 57% and 35%, respectively, vs 27% and 31% in the control group; *P*<.01), clarity with the use of the learning method (*Strongly agreed* and *Agreed* in the intervention group were 30% and 54%, respectively, vs 2% and 29% in the control group; *P*<.001), enough time to complete the proposed exercises (*Strongly agreed* and *Agreed* in the intervention group were 9% and 17%, respectively, vs 8% and 4% in the control group; *P*<.01), and improvement in the capacity to learn content (*Strongly agreed* and *Agreed* in the intervention group were 11% and 57%, respectively, vs 0% and 17% in the control group; *P*<.001). No difference was seen between the groups with respect to satisfaction regarding how the final assessment reflected the course syllabus (*Strongly agreed* and *Agreed* in the intervention group were 11% and 43%, respectively, vs 6% and 31% for the control group; *P*=.06).

**Table 4:** Effect of the different learning methods on the Profile of Mood States (POMS) total and subscale scores.

Mood state		Control group (n=48), mean (SD)		Intervention group (n=46), mean (SD)		*P* value^a^	
**Tension and anxiety**						.16	
	Baseline^b^		45.58 (8.43)		46.78 (9.70)		
	Postintervention		46.15 (9.45)		45.00 (10.09)		
	Difference		0.56 (8.88)		–1.78 (7.21)		
**Depression and melancholy**						.32	
	Baseline		47.38 (5.55)		47.39 (6.82)		
	Postintervention		49.21 (7.64)		47.93 (8.64)		
	Difference		1.83 (6.55)		0.54 (6.04)		
**Anger and hostility**						.05	
	Baseline		51.77 (9.52)		52.67 (8.95)		
	Postintervention		53.98 (11.79)		51.61 (10.24)		
	Difference		2.21 (7.97)		–1.07 (8.45)		
**Vigor and activity**						.67	
	Baseline		54.25 (7.30)		57.43 (6.05)		
	Postintervention		51.96 (7.55)		54.67 (6.77)		
	Difference		–2.29 (5.80)		–2.76 (4.82)		
**Fatigue and inertia**						.63	
	Baseline		47.75 (7.02)		46.59 (6.79)		
	Postintervention		48.73 (9.49)		46.80 (7.45)		
	Difference		0.98 (9.24)		0.22 (5.51)		
**Confusion and bewilderment**						.01	
	Baseline		38.54 (7.38 )		39.65 (8.02)		
	Postintervention		42.21 (8.43)		39.15 (7.64)		
	Difference		3.67 (8.66)		–0.50 (6.62)		
**Total score**						.09	
	Baseline		–17677.08 (2899.91)		–17565.22 (3453.13)		
	Postintervention		–18831.25 (4052.59)		–17582.61 (4088.16)		
	Difference		–1154.17 (3652.10)		–17.39 (2767.05)		

^a^Repeated-measures analysis of covariance (ANCOVA) was used to examine the differences between groups. Significance was set at *P*<.05; a trend toward significance was defined as .05≤*P*<.10.

^b^*Baseline* refers to the beginning of the 2-week study period.

The intervention group subjects used the app an average of 3.85 (SD 1.51) days per week. The *quiz* option was the most popular, used by 83% (38/46) of the subjects, followed by *word coupling* (29/46, 63%) and *alphabet soup* (20/46, 43%). In further analysis, the median number of days spent using the app per week (median 4 days) was used to categorize students as infrequent (<4 days/week) and frequent users (≥4 days/week). Motivation among the frequent users was significantly higher than among the infrequent users (IMMS_attention: frequent, mean score 50.04 [SD 5.17] vs infrequent, mean score 44.40 [SD 8.29], t_92_=–2.669, *P*=.01; IMMS_confidence: frequent, mean score 33.77 [SD 4.87] vs infrequent, mean score 29.30 [SD 4.38], t_92_=–3.222, *P*<.01; IMMS_satisfaction: frequent, mean score 24.00 [SD 3.03] vs infrequent, mean score 20.40 [SD 4.78], t_92_=–3.114, *P*<.01; and IMMS_total: frequent, mean score 142.85 [SD 15.52] vs infrequent, mean score 127.15 [SD 20.34], t_92_=–2.971, *P*<.01). No significant difference was detected, however, between the users in the subgroups in terms of IMMS_relevance (frequent, mean score 35.04 [SD 4.24] vs infrequent, mean score 33.05 [SD 5.92], t_92_=–1.328, *P*=.19). The frequent users also perceived their general linguistic competence in English to have significantly improved through the use of the app (*P*=.03), although this was not reflected when examining specific domains: written expression (*P*=.57), written comprehension (*P*=.18), and vocabulary (*P*=.11).

## Discussion

### Principal Findings

The blended learning method led to significant improvements in motivation, mood state, and satisfaction compared to traditional teaching. Moreover, frequent users of the app showed stronger motivation and perceived greater gains in their English-language competence than did infrequent users. However, the results suggest the intervention strategy provided no benefit in terms of knowledge absorbed. The latter finding is consistent with the results of two previous randomized controlled studies evaluating online learning methods [22,23]. In contrast, Chuang et al, whose work had a similar design to this investigation (ie, blended learning vs regular lecturing) and was of the same duration (ie, 2 weeks), reported significant differences in favor of blended learning [37]. It has been suggested that the use of audiovisual material, as employed in the latter study, might generate better learning outcomes [38].

### Comparison With Prior Work

In this work, motivation was measured by the validated IMMS tool. Higher scores were recorded for all motivation dimensions in the intervention group. Keller defines motivation as an innate characteristic of students, but also indicates that it can be influenced by external factors such as the instructional method used [39]. The innovative design for reviewing classes described in this work may be responsible for the increased motivation among the intervention group subjects. The use of augmented reality among middle school students [26], and of 3D computer environments among undergraduate students [40], have returned similar improvements in motivation. A recent study [41] reports that a mobile augmented reality (mAR) app boosted motivation in 35 students at four vocational education and training institutes over a period of 20 days. Real-time feedback, the degree of success achieved (ie, ranking), time on task (ie, frequent vs infrequent users), and learning outcomes (ie, theoretical tests) were some of the main predictors of improved learning in this latter study. The greatest difference between the subjects in the groups of this study was seen with respect to IMMS_attention (ie, the interest and curiosity of students in the learning process), which was significantly greater among the intervention subjects. The app's attractive interface, enjoyable quizzes, and user-friendliness may be partially responsible for this result.

The intervention group subjects appeared to be more involved in the learning process; they returned a significant change in two of the six mood state components assessed, which was reflected in the total score. Moreover, anger and hostility as well as confusion and bewilderment improved in the intervention group subjects, while these became worse (ie, increased scores) among those in the control group. The use of the app might thus have helped improve negative mood states. Pekrun et al [42] stated that academic achievement is a complex interaction between emotions, engagement, and performance, and this influences student self-appraisal. Flexible and creative learning strategies facilitate positive academic emotions, while more rigid strategies may spur negative emotions [42]. The “flow” concept proposes that an optimal educational experience may exist when students feel that a task is meaningful and, while perhaps challenging, that they are up to the challenge [43]. Other authors [17,44] who tested mAR interventions (ie, 30 minutes and 45 minutes in length), using a variation of the POMS questionnaire to measure change in mood state, only showed intrasubject changes.

Finally, satisfaction was greater among the intervention group subjects. It seems clear that the role of m-learning was perceived as an appropriate complement to traditional lecturing. With the development of advanced technologies, smartphones can be an effective learning tool for students. The possibility of reviewing classes whenever and wherever one likes, with tailored feedback (ie, scores for the games and quizzes), may have promoted the transfer of knowledge into their short-term and even long-term memories [45].

### Limitations

This study suffers from the limitation that it lasted only 2 weeks. This short time frame was due to external factors such as a funder-imposed deadline. Further, the iPOT app was created for use as a post-teaching period review tool, not as a study tool in itself. No difference was detected between the amount of knowledge absorbed by the members of the two groups. However, if, as the results show, the use of the app motivates students, a significant difference in what is learned might be expected after a longer period of use. Further work should investigate this.

### Conclusions

Students who received instruction via blended learning involving m-learning showed greater motivation, a better mood state, and greater satisfaction than those who received traditional lectures alone, although no difference was seen in terms of the amount of knowledge absorbed. Longer studies are needed to determine whether the improvements in these factors persist and whether they eventually translate into more knowledge being absorbed.

## Appendix

Multimedia Appendix 1 iPOT video tutorial.

Multimedia Appendix 2 CONSORT-EHEALTH checklist (V 1.6.1).

## Abbreviations

ANCOVA: analysis of covariance
ICC: intraclass correlation coefficient
IMMS: Instructional Materials Motivation Survey
mAR: mobile augmented reality
m-learning: mobile learning
OS: operating system
POMS: Profile of Mood States
QR: Quick Response
SSL: Secure Sockets Layer
TMD: Total Mood Disturbance
UCEES: Unit of Excellence on Exercise and Health
